# Autophagy Inhibition Potentiates the Anticancer Effects of a Bendamustine Derivative NL-101 in Acute T Lymphocytic Leukemia

**DOI:** 10.1155/2020/1520651

**Published:** 2020-02-14

**Authors:** Hang Gao, Siyue Lou, Huanwu Hong, Qiufu Ge, Huajun Zhao

**Affiliations:** ^1^College of Pharmaceutical Sciences, Zhejiang Chinese Medical University, Hangzhou 311402, China; ^2^Hangzhou Tinuo Pharmaceutical Co., Ltd., Hangzhou 310053, China

## Abstract

Acute T lymphocytic leukemia (T-ALL) is an aggressive hematologic resulting from the malignant transformation of T-cell progenitors. Drug resistance and relapse are major difficulties in the treatment of T-ALL. Here, we report the antitumor potency of NL-101, a compound that combines the nitrogen mustard group of bendamustine with the hydroxamic acid group of vorinostat. We found NL-101 exhibited efficient antiproliferative activity in T-ALL cell lines (IC_50_ 1.59–1.89 *μ*M), accompanied by cell cycle arrest and apoptosis, as evidenced by the increased expression of Cyclin E1, CDK2, and CDK4 proteins and cleavage of PARP. In addition, this bendamustine-derived drug showed both a HDACi effect as demonstrated by histone hyperacetylation and *p21* transcription and a DNA-damaging effect as shown by an increase in *γ*-H2AX. Intriguingly, we found that NL-101-induced autophagy in T-ALL cells through inhibiting Akt-mTOR signaling pathway, as indicated by an increase in LC3-I to LC3-II conversion and decrease of p62. Furthermore, inhibition of autophagy by 3-methyladenine increased apoptotic cell death by NL-101, suggesting a prosurvival role of autophagy. In summary, our finding provides rationale for investigation of NL-101 as a DNA/HDAC dual targeting drug in T-ALL, either as a single agent or in combination with autophagy inhibitors.

## 1. Introduction

Acute T-cell lymphoblastic leukemia (T-ALL) is a common malignant hematologic disease, accounting for 15% of children and 25% of adult ALL cases [1]. Chromosomal translocations, deregulated gene expression, duplications, deletions, and mutations of DNA are the main causes of T-ALL [2]. Combination chemotherapy can cure about 80% of child T-ALL cases, whereas adult patients with T-ALL have lower cure rate and a substantial proportion of patients develop resistance and finally relapse [3]. Thus, the search for novel agents that possess anti-T-ALL potency is of great significance.

Histone deacetylases (HDACs) are enzymes involved in the remodeling of chromatin and have been widely recognized as a promising target that can reverse aberrant epigenetic states associated with cancer [4, 5]. The HDAC inhibitor (HDACi) vorinostat was approved by U.S. Food and Drug Administration for treating cutaneous T-cell lymphoma [6]. Although many studies *in vitro* have shown that HDACi exerts substantially homogeneous and generally high cytotoxic responses in certain hematological malignancies, the overall response rate to HDACi in clinical settings is low in contrast with these *in vitro* assays. Both two typical HDACi tefinostat and vorinostat gained modest response in phase I study of the patients with advanced hematological malignancies [7, 8]. Thus, HDACi as single agent might be insufficient to achieve tumor regression and should be best exploited in combination protocols with other antitumor drugs.

Alkylating agent is a classic anticancer drug which induces apoptosis through activation of DNA damage stress responses, inhibition of mitotic checkpoints, and induction of mitotic catastrophe [9]. This class of agents has been used to treat metastatic breast cancer, gastric cancer, and several malignant hematological cancers [10]. However, systemic toxicity and cellular resistance impede their efficacy [11]. In clinical treatment of T-ALL, high doses of alkylating agents are recommended and are usually combined with multiple chemotherapeutic agents, which still do not improve the cure rate of T-ALL and are accompanied by recurrence, lethal neurotoxicity, and infectious complications [12–14].

Some studies have proven that combination of HDACi and alkylating agent exhibits potent anticancer activity [15, 16]. But the clinical data failed to obtain satisfactory results due to hematological toxicity [17]. Therefore, a novel DNA/HDAC dual targeting compound NL-101 that combines the nitrogen mustard group of bendamustine with the hydroxamic acid group of vorinostat was designed. It exerts a potent antitumor ability in multiple myeloma [18] and acute myeloid leukemia [19]. However, its activity in T-ALL was not investigated. In our study, we demonstrate that NL-101 can induce T-ALL cell death, cell cycle arrest, and DNA damage. More interestingly, it induces a prosurvival autophagy which may counteract drug efficacy. In general, our work proves that NL-101 is a promising compound against T-ALL and inhibition of autophagy can further improve its anticancer effect.

## 2. Materials and Methods

### 2.1. Cell Lines and Cell Culture

Jurkat and Molt-4 cell lines were purchased from the Cell Bank of the Institute of Biochemistry and Cell Biology, China Academy of Sciences (Shanghai, China), and stored in liquid nitrogen. CAM191 cell was purchased from Tongpai (Shanghai) Biotechnology Co., Ltd. Cells were cultured in 1640 medium (Gibco, USA) containing 10% fetal bovine serum (FBS, Gibco, USA) at 37°C in humidified incubator with 5% CO_2_.

### 2.2. Chemical and Regents

NL-101 was designed and synthesized by Hangzhou Minsheng Institute of Pharmaceutical Research. Dimethyl sulfoxide and 3-(4,5-dimethyl-2-thiazolyl)-2,5-diphenyl-2H-tetrazolium bromide (MTT) were purchased from Sigma-Aldrich (USA). Propidium iodide (PI)/RNase staining kit and Annexin V-FITC/7AAD kit were purchased from Becton Dickinson (San Diego, CA, USA). Acridine Orange base was purchased from Sigma-Aldrich (USA). Agarose was purchased from Invitrogen (USA). Low-melting agarose was purchased from Sangon Biotech (Shang Hai, China). Antibodies against p21 (ab18209), *γ*-H2AX (ab11134), were purchased from Abcam (Cambridge, UK). Antibody against acetylated histone H3 was from Merck Millipore. Antibodies against histone H3 (db972), H2AX (db5729), and Chk2 (db928) were from Diagbio, and antibodies against caspase-3 (#9662), cleaved caspase-3 (Asp175, #9664), caspase-8 (#4790), caspase-9 (#9508), cleaved caspase-9 (Asp330, #9501), PARP (#9532), c-Myc (#9402), cyclin E1 (#4129), Cdk2 (#2546), ATG5 (#2630), SQSTM1/p62 (#5114), LC3B (#3868s), p-mTOR (#5536), mTOR (#2938), p-Akt (#4060), Akt (#4691), p-Chk2 (#12302), and *β*-actin (#4970) were purchased from Cell Signaling Technologies (Danvers, MA, USA). And horseradish peroxidase-conjugated secondary antibodies were purchased from Bio-Rad (Hercules, CA, USA).

### 2.3. MTT Assay

The MTT assay was used to determine cell viability. Jurkat and Molt-4 cells were seeded into 96-well plates at a density of 10,000 cells/well and treated with different concentrations of NL-101 for 24, 48, and 72 h. Then, 20 *μ*L MTT solution (5 mg/ml) was added into each well and incubated for another 4 h. Formed formazan crystals were dissolved in 100 *μ*L DMSO and the absorbance was measured at 570 nm on a microplate reader (Bio-Tek, CA, USA). IC_50_ value was calculated by GraphPad Prism 5.0 software (Graph Pad Software, CA, USA).

### 2.4. Cell Cycle Detection

5 × 10^5^ cells were seeded in 6-well plates and treated without or with 2, 4 *μ*M NL-101 for 24 h, respectively. Then, cells were harvested, washed twice with ice-cold PBS, and fixed using 75% ethanol at −20°C overnight. Next, cells were washed again with ice-cold PBS, stained with PI/RNase (0.5 ml/test, 1 × 10^6^ cells) for 15 min at room temperature. All samples were analyzed by flow cytometer (Guava Technologies, Merck Drugs & Biotechnology, Germany), and DNA content was quantified using modfit software (Verity Software House, USA).

### 2.5. Cell Apoptosis Detection

Quantification of apoptotic cells was performed using the Annexin V-FITC Apoptosis Detection Kit. Briefly, 5 × 10^5^ cells were seeded in 6-well plates and treated with 2 *μ*M and 4 *μ*M NL-101 with or without 1 mM 3-MA. Then cells were harvested, stained with Annexin V-FITC/7-AAD, and then analyzed by flow cytometer.

### 2.6. AO Staining for Acidic Vesicular Organelles

5 × 10^5^ cells were seeded in 6-well plates and treated with 2 *μ*M NL-101 for 0, 4, 8, 12, and 24 h. Then, cells were harvested, washed twice with ice-cold PBS, and stained with 5 *μ*g/mL acridine orange for 15 min and examined under a fluorescence microscope (Nikon, Tokyo, Japan).

### 2.7. Comet Assay

Cells were seeded in 6-well plates. After treatment with NL-101 for 24 h, cells were harvested and resuspended at 1 × 10^5^ cells/ml density in ice-cold PBS. And then they were combined with low-melting agarose (1 g/100 ml in 1 × Tris Base, Boric, acid, EDTA) at a ratio of 1 : 10 (v/v) and spread on the comet slide. The slides solidified for 10 min at 4°C and then submerged in alkaline lysis buffer at 4°C overnight. Then slides were subjected to electrophoresis at 1.0 V/cm for 30 min. The slides were placed in 50%, 70%, and 100% ethanol and dried at 37°C for 15 min, respectively. The slides were then stained with Gel-Red (Beyotime, Shanghai, China) for 20 min in dark. Images were acquired using a fluorescent microscope.

### 2.8. Western Blotting Analysis

Cells were collected and lysed on ice in RIPA buffer supplemented with phosphatase inhibitors PMSF and protease inhibitors aprotinin. The protein concentration was determined by BCA protein concentration assay kit (Beyotime). Equal quantity of proteins was then separated on SDS-PAGE gels and transferred to PVDF membrane. The membranes were then blocked with 5% nonfat milk at room temperature for 1 h and incubated with the corresponding specific antibodies overnight at 4°C. Next, the membranes were washed three times with TBS-T (Tris-buffered saline-5% Tween 20) and incubated with the HRP-conjugated secondary antibody for 2 h at room temperature. Chemiluminescent detection was performed by ECL (BIO-RAD, USA). Anti-*β*-actin antibody served as a loading control.

### 2.9. RT-PCR

RNA was extracted with Trizol agent (Invitrogen, USA). Real-time PCR was performed in triplicate. Glyceraldehyde-3-phosphate dehydrogenase (GAPDH) was used as an internal control of RNA integrity. The primers used for qRT-PCR were as follows:

 
*p21* forward: 5′-TGCCGAAGTCAGTTCCTTGT-3′
 
*p21* reverse: 5′-CATTAGCGCATCACAGTCGC-3′
  GAPDH forward: 5′-AACCCTTAAGAGGGATGCTGC-3′
  GAPDH reverse: 5′-ATGAAGGGGTCGTTGATGGC-3′

### 2.10. Statistical Analysis

All data are expressed as mean ± SD. Statistical significance was analyzed using Student's *t*-test. The criterion of statistical significance was ^*∗*^*p* < 0.05; ^*∗∗*^*p* < 0.01; ^*∗∗∗*^*p* < 0.001.

## 3. Results

### 3.1. NL-101 Inhibits T-ALL Cell Proliferation *In Vitro*

**Figure 1:**
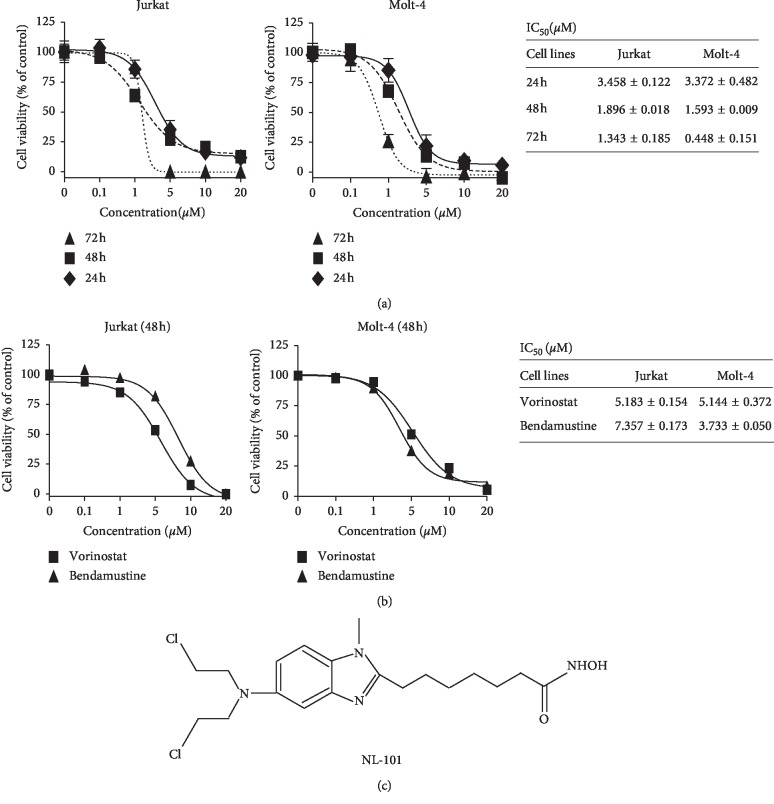
NL-101 inhibits proliferation of T-ALL in a dose- and time-dependent manner. T-ALL cells were seeded into 96-well plates with fresh RPMI-1640 medium with NL-101 (a), vorinostat, and bendamustine (b) at indicated concentrations. Cell viability was assessed by MTT analysis and IC_50_ values were calculated. Data are representative of three independent experiments. (c) The structure of NL-101.

To determine the effect of NL-101 on human T-ALL cells, Jurkat and Molt-4 cell lines were treated with different concentrations of NL-101 for 24, 48, and 72 h. MTT was used to evaluate cell viability and calculate the inhibition concentration at 50% (IC_50_) values. As shown in Figures 1(a) and 1(b), NL-101 significantly inhibited cell proliferation in a time- and dose-dependent manner. IC_50_ for Jurkat and Molt-4 cells at 48 h were 1.9 *μ*M and 1.6 *μ*M, respectively, while IC_50_ of bendamustine is 7.357 *μ*M and 3.733 *μ*M and vorinostat is 5.183 *μ*M and 5.144 *μ*M. These results suggested that NL-101 inhibits T-ALL cells proliferation superior to bendamustine and vorinostat. The structure of NL-101 is shown in Figure 1(c).

### 3.2. NL-101 Increases DNA Damage and Promotes Histone Acetylation Levels in Human T-ALL Cell Lines

**Figure 2:**
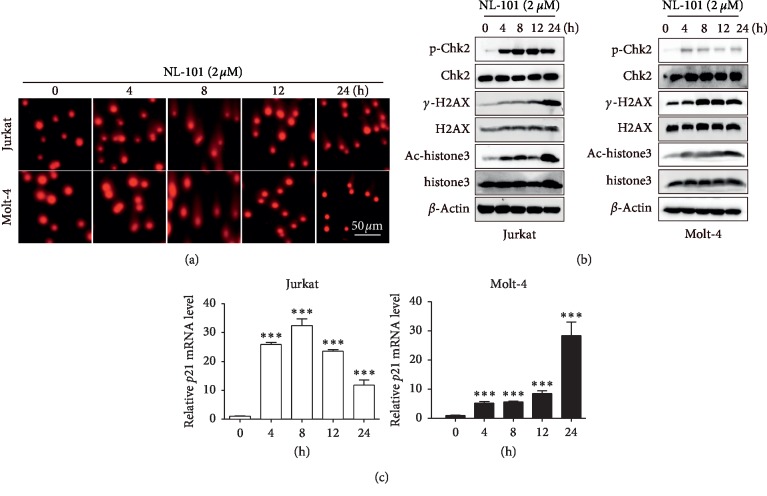
NL-101 induces DNA damage of T-ALL. (a) T-ALL cells were seeded into 6-well plates and cultured in the absence or presence of 2 *μ*M NL-101 for 4, 8, 12, and 24 h. Then, DNA damage was determined by comet assay described in Section 2.7. Scale bars, 50 *μ*m. (b) T-ALL cells treated with 2 *μ*M NL-101 for indicated times were subjected to western blotting analysis with specific antibody directed against p-Chk2, Chk2, *γ*-H2AX, H2AX, Ac-histone3, and histone H3. *β*-Actin was used as loading control. (c) The *p21* mRNA expression levels in T-ALL cells treated with NL-101 for different time points were determined by real-time PCR. Fold change in mRNA levels was calculated by normalizing to GAPDH. ^*∗∗∗*^*P* < 0.001. Data shows the representative of three independent experiments.

It is well known that alkylating agent plays an anticancer role by causing DNA damage. So, we hypothesize that NL-101 has similar effects. Both T-ALL cells were treated with or without NL-101 for 4 h to 24 h to detect DNA damage by comet assay. Our result showed that NL-101 treated cells present characteristic nuclei with tails due to separation of fragmented DNA (Figure 2(a)). Then DNA damage associated proteins were examined by western blotting analysis. As shown in Figure 2(b), NL-101 upregulated the phosphorylation of Chk2 and H2AX at Ser-139 residue (*γ*-H2AX) in a time-dependent manner.

**Figure 3:**
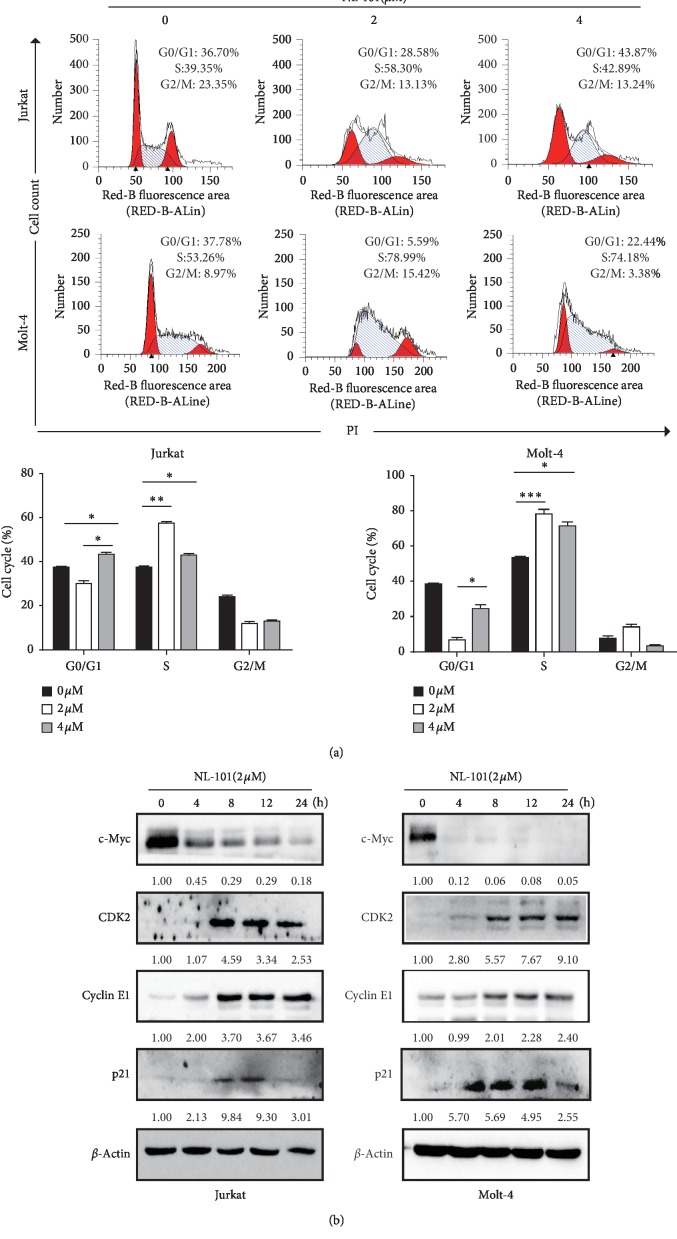
NL-101 induces cell cycle arrest at S phase in T-ALL. (a) The cell cycle distributions were examined in two T-ALL cells treated with NL-101 (0, 2, 4 *μ*M) for 24 h by staining with PI solution (left panel). Quantification of cell cycle progression was shown in the right panel. ^*∗*^*P* < 0.05; ^*∗∗*^*P* < 0.01; ^*∗∗∗*^*P* < 0.001. (b) Western blotting analysis of the expression of cell cycle-related proteins (c-Myc, CDK2, CyclinE1, and p21) in T-ALL cells. *β*-Actin was used as loading control. Data shows the representative of three independent experiments.

At the same time, in order to confirm the effect of NL-101 on histone acetylation levels, the expression of acetylated histone protein was detected. As expected, NL-101 strongly increased histone H3 acetylation after 4 h of treatment (Figure 2(b)). It has been reported that HDACi can induce accumulation of acetylated histones in the chromatin of the *p21* gene, which is associated with an increase in *p21* expression. Increased expression of *p21* plays a key role in the growth arrest [19]. Therefore, we analyzed the mRNA and protein expression of *p21*. We found that there was an increased transcription and expression level of *p21* among 4 h to 24 h after treatment of NL-101 (Figures 2(b) and 3(b)). These results indicated that DNA damage was involved in the anticancer activity of NL-101 and increased expression of *p21* induced by NL-101 was critical in the T-ALL cell death.

### 3.3. NL-101 Induces Cell Cycle Arrest at S Phase in T-ALL Cells

Loss of cell cycle control is one of the hallmarks of cancer [20] and based on the increased expression of p21, so we next evaluated whether NL-101 caused cell cycle arrest. Both cells were treated with 2 and 4 *μ*M NL-101 for 24 h. As shown in Figure 3(a), NL-101 arrested cells at S phase at 2 *μ*M while G0/G1 phase at 4 *μ*M. Consistent with the above results, western blotting analysis showed decreased expression of c-Myc and increased expression of CDK2, cyclin E1, and p21 in NL-101 treated cells (Figure 3(b)). These results suggested that the anti-T-ALL effect of NL-101 might be associated with the cell cycle arrest.

### 3.4. NL-101 Induces Cell Apoptosis in T-ALL Cells

**Figure 4:**
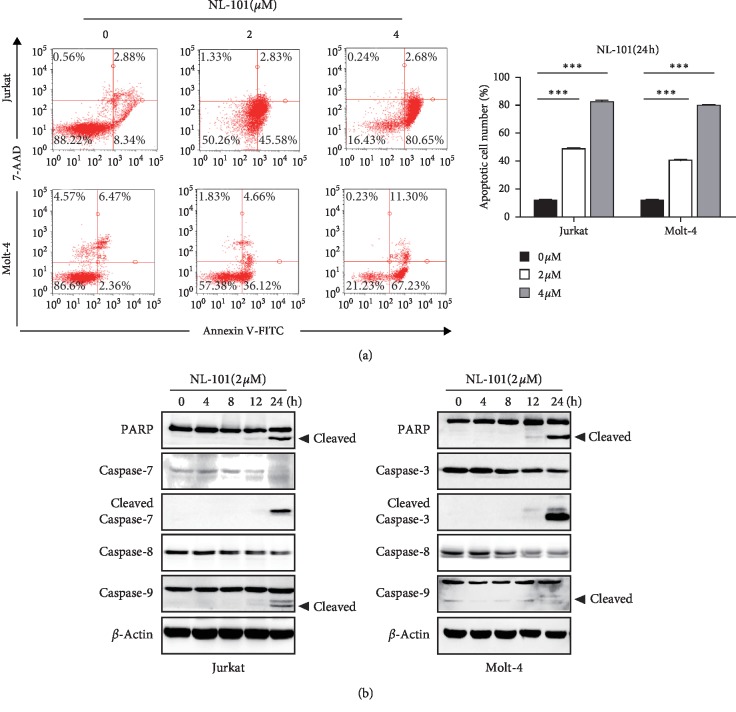
NL-101 induces apoptosis in T-ALL. (a) Cell apoptosis was determined after treatment with NL-101 using Annexin V/7-AAD double-staining assay by flow cytometry. The left panel shows representative micrographs of cell apoptosis and the right panel shows the quantification of apoptotic cells. ^*∗∗∗*^*P* < 0.001. (b) Western blotting analysis of apoptotic-related proteins such as the full length and cleavage of PARP, caspase-3/7/8/9. *β*-Actin was used as loading control. Data represent at least three independent experiments.

Apoptosis is a key part of the tumor-suppression mechanism [21], so we investigate whether NL-101 could induce apoptotic cell death. Both cell lines were treated with NL-101 and stained with Annexin V-FITC/7-AAD. As illustrated in Figure 4(a), NL-101 significantly induced cell apoptosis in a dose-dependent manner. 4 *μ*M of NL-101 resulted in 83% and 79% cell apoptosis in Jurkat and Molt-4 cells, respectively. Western blotting analyses showed the time-dependent cleavage of caspase-9/7 in Jurkat cells and cleavage of caspase-9/3 in Molt-4 cells. Simultaneous downregulation of caspase-8 expression and activated cleavage of PARP were also observed in both cells (Figure 4(b)). These results suggested that NL-101 inhibits T-ALL proliferation by activating intrinsic cell apoptotic pathway.

### 3.5. NL-101 Induces Cytoprotective Autophagy by Inhibiting mTOR Signaling Pathways in T-ALL Cells

**Figure 5:**
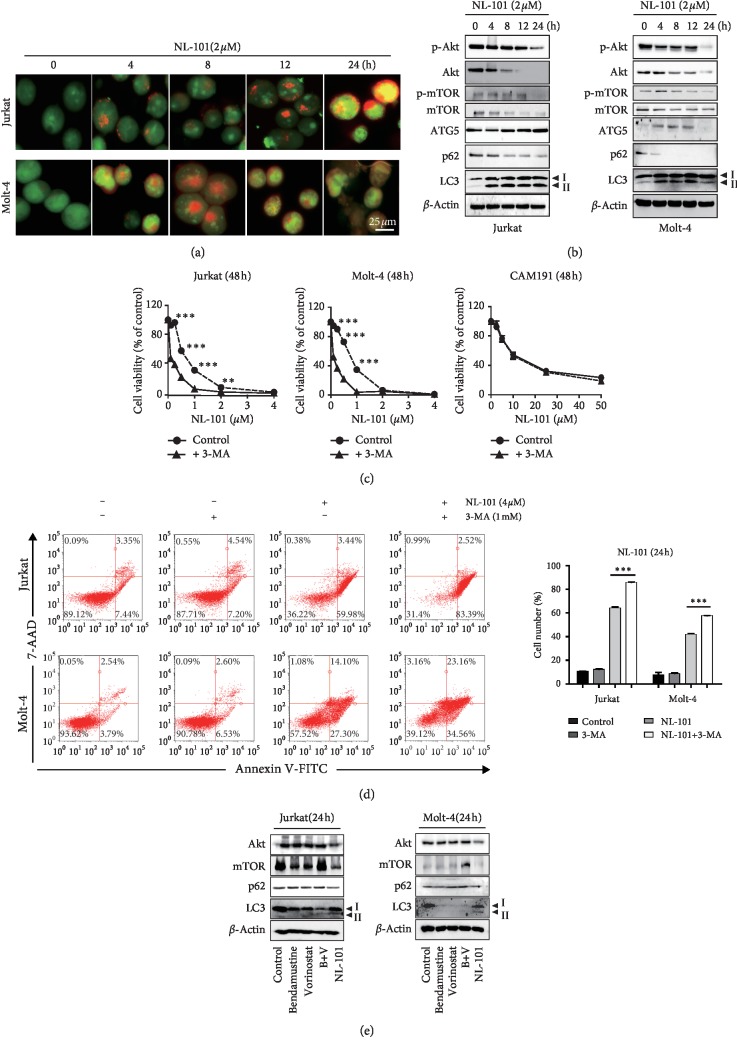
NL-101 induces prosurvival autophagy in T-ALL. (a) NL-101-treated T-ALL cells were stained with AO and examined under a fluorescence microscope. Scale bar, 50 *μ*m. (b) The expression level of autophagy-related protein after NL-101 treatment of T-ALL cells was assessed by western blotting analysis. *β*-Actin was used as loading control. (c) T-ALL cells and CAM191 cell were preincubated with or without 3-MA (1 mM) for 3 h and then incubated with indicated concentrations of NL-101, after which the cells were subjected to MTT assay (c), flow cytometry analysis of apoptotic cells (d), and western blotting analysis of LC3-II, PARP, and cleaved-caspase-3 (e). The expression level of autophagy-related protein after bendamustine (5 *μ*M), vorinostat (5 *μ*M), or a combination of bendamustine and vorinostat (B + V) and NL-101 (2 *μ*M) treatment of T-ALL cells were assessed by western blotting analysis. *β*-Actin was used as loading control. ^*∗∗*^*P* < 0.01; ^*∗∗∗*^*P* < 0.001. Data represent at least three independent experiments.

Autophagy exerts multiple roles in cell death progress. Thus, to investigate whether autophagy contributes to the anti-T-ALL effects of NL-101, we first detected the occurrence of autophagy by AO staining. As shown in Figure 5(a), NL-101 caused the formation of acidic autophagic vesicles. Consistently, western blotting data showed that NL-101 increased the conversion of LC3-I into LC3-II, upregulated the expression of ATG5, and downregulated the expression of p62 (Figure 5(b)), an event preceding the cleavage of caspase and PARP. Since studies have reported that the Akt/mTOR signaling pathway is a vital negative regulator of autophagy [22], we then explored whether NL-101 inhibited mTOR signaling pathway to induce autophagy in T-ALL cells. The results showed that NL-101 reduced the phosphorylation of Akt and mTOR (Figure 5(b)), suggesting that NL-101 can induce autophagy by inhibiting Akt/mTOR signaling pathway. To further clarify the role of NL-101-induced autophagy in T-ALL, we used 3-MA to inhibit autophagy at an early stage with or without combination of NL-101. MTT results revealed that inhibition of autophagy further reduced cell viability (Figure 5(c)), while dramatically increasing apoptotic cell ratio (Figure 5(d)). Meanwhile, the results showed that the effect of combined therapy on normal lymphocytes was less than T-ALL. These results implied that NL-101 activated cytoprotective autophagy and the use of autophagy inhibitors will potentiate the anti-T-ALL effect of NL-101. Moreover, Akt/mTOR signaling pathway was not inhibited by vorinostat or bendamustine alone, which indicated that the induction of autophagy was a special trait of NL-101 (Figure 5(e)).

## 4. Discussion

T-ALL is an aggressive disease characterized as extramedullary infiltration of lymph nodes and other organs such as CNS and the presence of a mediastinal mass arising from the thymus [23]. Although some treatment has gained favorable outcomes, relapsed T-ALL especially in adults is common [24]. Even in the presence of standard chemotherapy, only 30% to 40% of patients response and the responder's median OS is 6 months [25]. Thus, a novel strategy on the treatment of T-ALL is urgently required. HDACi vorinostat is used to treat cutaneous T-cell lymphoma (CTCL), while another HDACi panobinostat was used for multiple myeloma [26]. In contrast to high cytotoxic response *in vitro*, the effect *in vivo* is not ideal. In a retrospective analysis of advanced leukemia and myelodysplastic syndromes, the existence of ROS scavenger may result in resistance to HDACi [7]. Another possible reason limiting the application of HDACi is that it can activate autophagy, thus helping cancer cells to defeat unfavorable stress [27]. To overcome these problems, combination therapy is considered. Since HDACi can make chromatin more relaxed, it can enhance cell sensitivity to alkylating agent [28]. We noticed that there are some relevant studies reporting the exciting results of the combination of these two kinds of drugs, exhibiting favorable synergy *in vitro* [24, 29, 30]. However, serious hematological toxicity was also observed [17]. Moreover, such a combination scheme is only limited in the application of myeloma and the data *in vivo* are not sufficient. Since the bendamustine-derived drug NL-101 exhibited significantly enhanced anticancer activity in multiple myeloma, we hypothesized the new compound may have potential effect in T-ALL. As expected, NL-101 exhibited superior anticancer ability *in vitro* compared to bendamustine and vorinostat, suggesting this kind of structural modification is beneficial to improve the anticancer efficacy.

It is widely recognized that epigenetic abnormalities including posttranslational modifications of histones acetylation play a pivotal role in cancer development and progression [31]. Here, H3 acetylation level can be upregulated by NL-101. The results indicated that NL-101 may have great potential in cancer treatment with epigenetic histone modifications via reestablishing the cellular acetylation homeostasis [32]. In addition, since DNA damage caused by alkylating agent is involved in the oncogenesis process [28, 33, 34], we investigated NL-101's effect on DNA damage as well. Our result showed that NL-101 could upregulate the phosphorylation on Serine 139 of the histone variant H2AX (*γ*-H2AX), which is the hallmark protein of DNA damage response. Indeed, there are various studies reporting HDACi exhibits synergistic effects with alkylating agents on growth inhibition of MM cells and glioblastoma cells [15, 16, 35]. In addition to promoting apoptosis and inducing cell cycle arrest, it will enhance DNA damage response associated with enhanced mitotic catastrophe, which is useful for us to further study on NL-101. There are some explanations for these synergistic benefits. One possible reason is that DNA will be more accessible to alkylating agents because of loose chromatin conformation induced by HDACi; another speculation is that HDACi will change the expression of genes involved in DNA damage responses. These hypotheses possibly explain why after structural transformation, NL-101 has stronger anticancer ability in contrast to single drug used alone.

Many researches have reported that HDACi can induce G1 phase arrest with reduction of c-Myc and induction of p21 [30, 36–38]. It is clear that c-Myc expression levels tightly relate to cell proliferation. Many reports show c-Myc downregulation inhibits cell cycle progression through regulating transcriptional activation of target genes such as Cyclin D2 and Cyclin E1 and E2F1, while stimulating p21 activity and suppressing DNA replication [39–41]. In our study, NL-101 arrested cells at G0/G1 phase at higher concentration while inducing S phase arrest at lower concentration; this was accompanied by reduction of c-Myc and induction of CDK2, Cyclin E1, and p21. We speculate that NL-101-induced cell cycle arrest at different stages is possibly due to its “alkylation activity,” exhibiting similar concentration-dependent properties as bendamustine. Indeed, a considerable part of research has found that bendamustine alone can induce S phase arrest in different cell lines due to inducing DNA strand breaks [15, 42]. Moreover, Beeharry et al. found that in a wide panel of cancer cell lines, bendamustine presented concentration-dependent effects on cell cycle checkpoint [33]. However, the underlying mechanism of NL-101's concentration-dependent cell cycle arrest still remains to be explored.

In this study, we also focused on NL-101's role in autophagy. Autophagy is considered to have conflicting effects on cancer development. It may constitute a mechanism utilized by tumor cells to survive hypoxic, metabolic, and chemotherapeutic stress. There are several studies reporting that inhibiting certain autophagy genes or blocking the autophagy by inhibitors can enhance the sensitivity to chemotherapy [43, 44]. However, autophagy also potentiates the anticancer effects even with the same drug [45]. This may be associated with the state of cancer development and the type of cancer. In our study, we analyzed the acidic vesicular organelles (AVOs) and cells treated with NL-101 resulted in formation of yellow-orange AVOs. The autophagy-related protein expression also confirmed that the molecular mechanism is through inhibition of Akt/mTOR signaling. Interestingly, vorinostat or bendamustine alone failed to induce autophagy [29, 46], thus suggesting that the induction of autophagy is a special trait of NL-101. Generally speaking, Akt needs phosphorylation of threonine phosphorylation site (thr308) or serine phosphorylation site (ser473) to regulate cell's function. The phosphorylation of Akt activates its substrate, rapamycin target protein (mTOR), either directly by phosphorylating mTOR or indirectly by inactivating tuberous sclerosis complex 2 (TSC2) [47], to maintain Rheb's GTP binding state and then enhance the activation of mTOR [48]. Surprisingly, a decrease in total mTOR and Akt protein level was also observed in both cell lines treated by NL-101 and this occurred earlier than that of phosphorylation protein. Recent study showed that [49–51] ubiquitination can target proteasomal degradation of mTOR and other proteins, leading toward induction of autophagy and apoptosis. This inspired us to speculate that NL-101 activated protein ubiquitination associated with proteasome degradation of Akt and mTOR to induce autophagy, rather than simply affecting their phosphorylation level. Further experiments should be done to validate this hypothesis. Further investigation indicated that NL-101-induced autophagy is cytoprotective. When autophagy is blocked by inhibitor 3-MA, the ratio of apoptotic cells increased, suggesting that inhibiting autophagy may enhance the T-ALL sensitivity to NL-101. In our study, one interesting point is that autophagy occurs earlier than apoptosis. To some extent, it is also seen as a manifestation of resistance to drug.

There are extensive *in vitro* and *in vivo* studies confirming that combining autophagy inhibitors and chemotherapeutic drugs are an effective way to improve anticancer efficiency. A clinical trial of glioblastoma shows that patients treated with CQ inhibiting the lysosome and alkylating agent temozolomide can prolong median survival [52]. Furthermore, autophagy-related genes (ATGs) knockdown or treated with CQ also reduce viability of both epirubicin-sensitive and epirubicin-resistant triple-negative breast cancer cells [53]. Another lysosome inhibitor HCQ also achieved gratifying results when combined with vorinostat in a phase I human study, exhibiting good tolerability in solid tumors. Other similar human trials were also carried out in melanoma, glioblastoma, and spontaneously occurring lymphoma [54–57]. However, there are some arguments against inhibiting autophagy in cancer therapy. These studies suggest that autophagy inhibition will reduce antitumor T-cell responses [58]. Thus, it still remains a complex question as to exactly what kind of role autophagy plays in cancer progression.

In summary, our study shows that NL-101, a bendamustine-derived DNA/HDAC dual targeting compound, could inhibit T-ALL proliferation *in vitro* through inducing DNA damage, arresting the cell cycle, and activating caspase-dependent apoptosis. In addition, NL-101 could induce prosurvival autophagy through inhibiting the Akt/mTOR signaling pathway; the combination with autophagy inhibitor 3-MA could improve its anticancer efficiency. Our results provide a novel perspective for T-ALL therapy and support the clinical evaluation of this agent in T-ALL patients.

## 5. Conclusion

Our findings provide rationale of NL-101 as a DNA/HDAC dual targeting drug in T-ALL, either as a single agent or in combination with autophagy inhibitors.

## Data Availability

The data used to support the findings of this study are available from the corresponding author upon request.
